# Mental health status and its associated factors among female nurses in the normalization of COVID-19 epidemic prevention and control in China

**DOI:** 10.3389/fpubh.2022.1088246

**Published:** 2023-01-06

**Authors:** Xiaofei Mao, Wei Dong, Jianguo Zhang, Fan Zhang, Wenxi Deng, Ziqiang Li, Tianya Hou

**Affiliations:** Faculty of Psychology, Naval Medical University, Shanghai, China

**Keywords:** mental health, China, COVID-19, associated factor, female nurse, anxiety, depression

## Abstract

**Objective:**

To investigate mental health status and its associated factors among female nurses in the normalization of COVID-19 epidemic prevention and control in China.

**Methods:**

Random cluster sampling was applied to recruit 740 female nurses in China. The respondents completed the survey with mobile devices. Demographic questionnaire, Generalized Anxiety Disorder-7, Patient Health Questionnaire-9, Insomnia Severity Index, and The Impact of Event Scale-Revised were used to assess demographic Information, anxiety, depression, insomnia and PTSD symptoms, respectively. The associated factors of mental health status were identified by binary logistic regression analysis.

**Results:**

The prevalence of anxiety and depression was 7.9 and 17.8%, respectively. Insomnia was an associated factor of anxiety (OR = 6.237, 95%CI = 6.055–23.761, *P* < 0.001) and depression (OR = 9.651, 95%CI = 5.699–22.370, *P* < 0.001), while PTSD was an associated factor of anxiety (OR = 11.995, 95%CI = 2.946–13.205, *P* < 0.001) and depression (OR = 11.291, 95%CI = 6.056–15.380, *P* < 0.001), Being married was a protective factor of depression (OR = 0.811, 95%CI = 1.309–6.039, *P* < 0.01).

**Conclusion:**

Female nurses showed problems in mental health. Insomnia, PTSD and marital status were associated with mental health. The hospital management should pay more attention to the unmarried groups, and strive to improve the sleep quality of female nurses and reduce their stress caused by traumatic events.

## Introduction

Previous studies indicate that nurses faced a wide range of stressors, such as the huge workload caused by the high requirements of the tense system, shift responsibility, work-family conflict, etc. (1), which makes them, especially high-risk nurses, more vulnerable to develop mental problems (2). The outbreak of coronavirus disease 2019 (COVID-19) has arisen mounts of psychological problems to the health care workers in China, especially nurses. They experienced high mental burden and reported more severe degrees of mental health symptoms (3–6). According to the study conducted by Lai et al. the nurses were reported to have a significantly higher level of depression than the physicians do during the COVID-19 (7). Moreover, a study focusing on mental health of medical staff in Xinjiang province of China found nurse were more likely to show psychological problems than clinicians (8).

With the effort of Chinese government, the epidemic situation of COVID-19 has been effectively controlled, but the epidemic situation is still sporadic. China entered the stage of normalized epidemic prevention and control since May 2020 (9). China classified all counties as low-risk for COVID-19 from May 7, 2020, since no domestic cases had been reported on the Chinese mainland for four consecutive days as of May 6, with no new deaths for 22 consecutive days. Correspondingly, the national epidemic prevention and control policy has been changed from the blockade policy at the beginning of the outbreak into the normalization of COVID-19 epidemic prevention and control (10). The general policy is to prevent external input and internal rebound, insist on timely discovery, rapid disposal, precise control and effective treatment. That is, compared with many other countries that are starting to lift restrictions that were first imposed 2 years ago in order to slow the spread of COVID-19 (11), China still has COVID-19 restrictions in the normalization of COVID-19 epidemic prevention and control, such as Regular nucleic acid testing. This has imposed a huge burden on healthcare systems.

Though the severe situation faced by nurses has changed, they still worked under great pressure. It is important to understand the status of mental health of nurse group and associated factors. However, the work concerning the mental health of Chinese female nurses in the normalization of COVID-19 epidemic prevention and control is still missing. Recently, a cross-sectional study investigated the mental health status and its potential impact factors among male and female nurses from low-risk areas under normalized COVID-19 pandemic prevention and control in Jiangsu province, China (11). Prior reports have shown females are more vulnerable to poor mental health problems than males (12–14), and gender differences have been found regarding the influencing factors and influencing factors of mental health (15–17). Besides, female nurses accounted for the vast majority of nurses in China. Harding et al. found that the percentage of female nurses was 91% in New Zealand, 90.4% in the United States, 89% in UK, 88.3% in Australia and 77% in the Netherlands (18). The percentage of Chinese female nurses was 98% in 2017, which is higher compared with that in developed countries (19). However, studies concerning the metal health of Chinese nurses during the period of COVID-19 and normalization of COVID-19 epidemic prevention and control didn't distinguish the gender differences when it comes to the mental health and its associated factors, leading to a consequence that the status of mental health and its associated factors of female Chinese nurses remain still unclear.

Therefore, we aimed to conduct a cross-sectional study to clarify the status of mental health and its associated factors among female nurses in the normalization of COVID-19 epidemic prevention and control in China.

## Methods

### Participants and procedures

A cross-sectional study was conducted in January 2022. G^*^Power software version 3.1.9.7 was used to estimate the required sample size of this study. The present study used binary logistic regression analysis to analyze the association between mental health and associated factors. Therefore, F-test (Linear multiple regression: Fixed model, *R*^2^ increase) was employed. Effect size (*f*^2^) was set at 0.15 and alpha value was set at 0.05. Approximately 189 participants would provide 95.07% power to detect a statistical significance.

There were 3 inclusion criteria in this study. They were listed as follows, I. no dyslexia, II. 18 years old or above, and III. working in hospital under the normalization of COVID-19 epidemic prevention and control. The exclusion criterion was that female nurses had a history of mental illnesses. Convenience sampling was applied to recruit participants. According to the inclusion and exclusion criteria, 740 female nurses were recruited from 5 hospitals in Jiangsu Province of China with the efforts of members from the research team. The normalization of COVID-19 epidemic prevention and control in Jiangsu Province is same as other parts of China. Before filling out the online questionnaires, participants were asked if they were willing to take part in the study. Only those who volunteered to this research signed papery informed written consent. Respondents filled out all the scales in a Chinese version of questionnaire website called Wenjuanxing (https://www.wjx.cn/). The questionnaires can only be submitted after all the questions have been answered. All the data was collected *via* their smart mobile phone.

Ethical approval was obtained from the Naval Medical University before the initiation of the research project. Participants were assured their responses were anonymous and confidential. Participants were free to withdraw at any time without penalty.

### Measures

#### Demographics

In the present study, demographic information including age, years of working, medical isolation, night shift last month, vaccine against COVID-19, marital status, professional title, employment type, child status, weekly hours of working and working department were recorded.

Age was divided into two groups (20–22) [younger group ( ≤ 30 years old) and middle-age group (>30 years old)]. Years of working was divided in three groups ( ≤ 5, 6–10, >10). Medical isolation was categorized as having been isolated or not. The number of night shift last month was divided into two groups (< 4, ≥4). Vaccine against COVID-19 was categorized into being vaccinated or not. Marital status was divided into married or unmarried (single, divorced or widowed). Professional title was divided into 2 groups (junior title, intermediate or senior title). Employment type was coded as permanent contract employee or fixed-term contract employee. Child status was categorized into no child and having at least one child. Hours of working per week was divided into two groups ( ≤ 40, >40). Working departments were grouped into high-risk and low-risk units. Nurses working in fever clinics, COVID-19 medical unit and emergence department were considered as high-risk people, while the others were identified as low-risk nurses (23).

#### Generalized anxiety disorder-7

The GAD-7 is a valid and efficient tool for screening anxiety and assessing its severity in clinical practice and research (15). The 7-item questionnaire is used to ask participants how often they are bothered by each symptom during the last 2 weeks. Response options are “not at all” “several days” “more than half the days” and “nearly every day” scored as 0, 1, 2, and 3, respectively. Cut-off scores of 5, 10 and 15 are classified as mild, moderate and severe anxiety (24). Respondents with moderate or severe anxiety are suspected of having anxiety. In the present study, the Cronbach's alpha was 0.960.

#### Patient health questionnaire-9

The PHQ-9 includes 9 items pertaining to the DSM-IV criteria for depressive disorder (25). Each item is rated on a 4-point Likert scale from 0 to 3 (0-never; 1- several days; 2-more than half the time; and 3-nearly every day) within the last 2 weeks before the completion of the survey. Cut-off scores of 5, 10 and 15 are classified as mild, moderate and severe depression (26). Respondents with moderate or severe anxiety are suspected of having depression. In the present study, the Cronbach's alpha was 0.935.

#### Insomnia severity index

ISI is a brief self-assessment tool, which has been previously proven as a reliable and valid instrument to quantify perceived insomnia severity (27). It included 7 items, and each item can be rated using a 5-point Likert scale, ranging from 0 to 4. A Higher score indicated a higher severity of insomnia. The total score ranges from 0 to 28. The cut-off score of 8 is defined as the presence of insomnia (28), indicating subjects may have sleep difficulties. In the present study, the Cronbach's alpha was 0.926.

#### The impact of event scale-revised

The IES-R was used to assess posttraumatic stress symptoms caused by traumatic events. The IES-R scale includes 22 items and consists of three subscales: intrusiveness, avoidance and hyperarousal. The total scores of the scale ranges from 0 to 88. An IES-R total score >33 is identified as having PTSD symptoms (29), suggesting participants may have traumatic experience. In the present study, the Cronbach's alpha was 0.976.

### Statistical analysis

Data were analyzed with IBM SPSS (Version 21.0). The significance level was set at α = 0.05, and all tests were 2-tailed. Kolmogorov-Smirnov test was applied to check whether the data of anxiety and depression conform to normal distribution. The results shown that the total scores of anxiety and depression weren't normally distributed (all *P* < 0.001). Therefore, Mann-Whitney U-test was used to compare the differences for categorical variables with two groups and the Kruskal-Wallis test was used when having more than two groups. Binary logistic regression analysis was conducted for detecting the associated factors of anxiety and depression.

## Results

### Demographic characteristics

A total of 740 female nurses were recruited in the present study with the average age of 30.53 ± 6.65 years old. Demographic Characteristics were listed in Table 1. Most participants were aged 30 or below [433 (58.5%)], had no medical isolation experience [667 (90.1%)], had more than 4 night shifts last month [422 (57.0%)], had been vaccinated with COVID-19 vaccine [681 (92.0%)], were married [473 (63.9%)], had a junior professional title [473 (63.9%)], were fixed-term employees [647 (87.4%)], had at least 1 child [413 (55.8%)], worked in low risk units [662 (89.5%)], worked < 40 h per week [520 (70.3%)]. 194 (26.2%) of the participants reported PTSD symptoms, 61 (8.2%) of them had sleep difficulties.

**Table 1:** Characterization and distribution of anxiety and depression.

**Variables**	**Respondents**	**Anxiety**			**Depression**		
	***N*** **(%)**	**M** ±**SD**	**Z /**χ^2^	***P***	**M** ±**SD**	**Z /** χ^2^	***P***
**Age**							
Younger group ( ≤ 30)	433 (58.5%)	3.37 ± 4.04	−2.17	0.030	5.96 ± 5.24	−1.73	0.084
Middle-aged group (>30)	307 (41.5%)	3.95 ± 4.08			6.50 ± 5.02		
**Medical isolation**							
Yes	73 (9.9%)	3.30 ± 3.95	−0.86	0.390	5.57 ± 5.14	−0.80	0.421
No	667 (90.1%)	3.64 ± 4.07			6.23 ± 5.15		
**Night shifts last month**							
< 4	318 (43.0%)	3.10 ± 3.78	−2.76	0.006	5.46 ± 4.89	−3.43	0.001
≥4	422 (57.0%)	4.00 ± 4.22			6.73 ± 5.28		
**Vaccine shots**							
Yes	681 (92.0%)	3.63 ± 4.03	−0.70	0.487	6.21 ± 5.14	−0.51	0.614
No	59 (8.0%)	3.42 ± 4.38			5.92 ± 5.27		
**Years of working**							
1–5 years	268 (36.2%)	3.68 ± 4.13	3.92	0.141	6.25 ± 5.32	1.78	0.410
6–10 years	242 (32.7%)	3.25 ± 3.86			5.83 ± 4.94		
>10 years	230 (31.1%)	3.92 ± 4.17			6.50 ± 5.16		
**Marital status**							
Unmarried	267 (36.1%)	3.68 ± 4.03	−0.62	0.536	6.61 ± 5.41	−1.58	0.114
Married	473 (63.9%)	3.58 ± 4.08			5.95 ± 4.99		
**Professional title**							
Junior	473 (63.9%)	3.41 ± 4.00	−2.16	0.031	6.00 ± 5.19	−1.53	0.126
Intermediate and senior	267 (36.1%)	3.99 ± 4.14			6.51 ± 5.07		
**Employment type**							
Permanent	647 (87.4%)	4.09 ± 4.24	−1.33	0.185	6.47 ± 5.07	−0.79	0.428
Fixed-term	93 (12.6%)	3.55 ± 4.03			6.15 ± 5.16		
**Child status**							
No child	327 (44.2%)	3.54 ± 3.96	−0.23	0.822	6.31 ± 5.33	−0.33	0.740
Have children	413 (55.8%)	3.68 ± 4.14			6.09 ± 5.01		
**Working department**							
High-risk units	78 (10.5%)	4.45 ± 4.29	−1.92	0.055	6.74 ± 4.87	−1.24	0.217
Low-risk units	662 (89.5%)	3.52 ± 4.02			6.12 ± 5.18		
**Weekly hours of working**							
≤ 40 h	520 (70.3%)	3.31 ± 3.97	−3.75	< 0.001	5.78 ± 5.11	−3.93	< 0.001
>40 h	220 (29.7%)	4.34 ± 4.19			7.14 ± 5.13		
**PTSD**							
Yes	194 (26.2%)	7.37 ± 4.12	−14.42	< 0.001	10.64 ± 4.99	−13.80	< 0.001
No	546 (73.8%)	2.28 ± 3.09			4.60 ± 4.19		
**Insomnia**							
Yes	61 (8.2%)	7.56 ± 4.92	−7.06	< 0.001	12.15 ± 5.32	−8.64	< 0.001
No	679 (91.8%)	3.26 ± 3.78			5.65 ± 4.79		

Younger group showed significantly higher scores in anxiety and depression (*P* = 0.084, marginal significance) than older group. Those who worked on night shift more than 4 times last month had significantly higher levels of anxiety and depression. Female nurses with junior professional titles reported significantly serious anxiety. The ones who worked in high risk units had higher scores of anxiety (*P* = 0.055, marginal significance). Female nurses who worked more than 40 h per week reported a significantly higher level of anxiety and depression. Besides, nurses with PTSD symptoms or insomnia reported significantly higher scores of anxiety and depression compared to those without.

### Mental health status and its associated factors

The average score of GAD-7 was 3.61 ± 4.06 with 53 (7.2%) and 5 (0.7%) female nurses reporting moderate and severe anxiety respectively, 234 (31.6%) respondents showing mild anxiety. Therefore, 7.9% of our participants were suspected of having symptoms of anxiety. The average score of PHQ-9 was 6.19 ± 5.15. 74 (10.0%) and 58 (7.8%) female nurses reporting moderate and severe depression respectively, 310 (41.9%) respondents showing mild anxiety. Thus, 132 (17.8%) female nurses may have symptoms of depression. In order to explore the associated factors influencing the metal health status of female nurses, binary logistic regression analysis was carried out.

The results of binary logistic regression analysis listed in Table 2 showed that female nurses who reported a higher level of PTSD symptoms had a higher level of anxiety [OR = 6.237, 95%CI = (2.946–13.205), *P* < 0.001] and depression [OR = 9.651, 95%CI = (6.056–15.380), *P* < 0.001]. Besides, having insomnia was associated with a higher level of anxiety [OR = 11.995, 95%CI = (6.055–23.761), *P* < 0.001] and depression [OR = 11.291, 95%CI = (5.699–22.370), *P* < 0.001]. Marital status was also an associated factor influencing female nurses' depression. Married female nurses had a lower level of depression [OR = 0.811, 95%CI = (1.309–6.039), *P* < 0.01).

**Table 2:** Associated factors of mental health status identified by binary logistic regression analysis.

**Variables**	**Anxiety**			**Depression**		
	**OR**	**95% CI**	***P***	**OR**	**95% CI**	***P***
**Age**						
Younger group ( ≤ 30)	1 [Reference]			1 [Reference]		
Middle-aged group (>30)	0.835	0.251–2.785	0.770	0.703	0.282–1.752	0.449
**Medical isolation**						
Yes	1 [Reference]			1 [Reference]		
No	1.526	0.569–4.092	0.401	0.804	0.366–1.765	0.586
**Night shifts last month**						
< 4	1 [Reference]			1 [Reference]		
≥4	0.813	0.418–1.584	0.543	0.728	0.449–1.181	0.198
**Vaccine shots**						
Yes	1 [Reference]			1 [Reference]		
No	1.287	0.398–4.164	0.674	1.277	0.529–3.084	0.587
**Years of working**						
1–5 years	1 [Reference]			1 [Reference]		
6–10 years	1.673	0.362–7.722	0.510	1.054	0.351–3.159	0.926
>10 years	0.775	0.249–2.416	0.661	1.046	0.446–2.453	0.918
**Marital status**						
Unmarried	1 [Reference]			1 [Reference]		
Married	0.807	0.295–2.208	0.676	0.811	1.309–6.039	< 0.01
**Professional title**						
Junior	1 [Reference]			1 [Reference]		
Intermediate and senior	1.033	0.335–3.181	0.955	1.377	0.599–3.166	0.452
**Employment type**						
Permanent	1 [Reference]			1 [Reference]		
Fixed-term	0.684	0.242–1.937	0.475	1.442	0.676–3.075	0.343
**Child status**						
No child	1 [Reference]			1 [Reference]		
Have children	0.651	0.207–2.048	0.463	0.853	0.352–2.070	0.725
**Working department**						
High-risk units	1 [Reference]			1 [Reference]		
Low-risk units	1.315	0.555–3.113	0.534	0.524	0.239–1.151	0.108
**Weekly hours of working**						
≤ 40 h	1 [Reference]			1 [Reference]		
>40 h	0.473	0.166–1.349	0.161	0.761	0.404–1.433	0.397
**PTSD**						
Yes	1 [Reference]			1 [Reference]		
No	6.237	2.946–13.205	< 0.001	9.651	6.056–15.380	< 0.001
**Insomnia**						
Yes	1 [Reference]			1 [Reference]		
No	11.995	6.055–23.761	< 0.001	11.291	5.699–22.370	< 0.001

## Discussion

As far as we know, this is the first study concerning the female nurses' mental health status and its associated factors in the normalization of COVID-19 epidemic prevention and control in China. In the present cross-sectional study, we found 7.9% and 17.8% of our participants showed anxiety and depression symptoms, respectively. Furthermore, we found that insomnia, PTSD and marital status were associated factors affecting anxiety and depression.

The outbreak of COVID-19 has arisen a large number of psychological problems to the health care workers in China (8, 30, 31). A review focusing nursing population from December 2019 to March 2020 found that their mental health status was severe (32). Lai et al. conducted a survey about mental health outcomes by GAD-7 and PHQ-9 among health care professionals working with coronavirus-19 patients. According to their findings, 12.7 and 15.5% of the Chinese nurses reported anxiety and depression, respectively (7). In another study by Que et al., 14.9% of the Chinese nurses showed anxious symptoms (GAD-7), and 12.02% of them had depressive symptoms (PHQ-9) (33). Thus, compared with studies during the COVID-19 epidemic from China, the level of anxiety of female nurses in our study was lower than that reported by prior research, whereas the level of depression was higher. When comparing our findings with the findings from other countries, the mental health condition of female nurses in the normalization of COVID-19 epidemic prevention and control of China was better than those of other countries in the period of COVID-19. For example, 21.4% of Japanese nurses involved with COVID-19 patients showed anxiety and 19.7% of them had depression (34). 43 and 26% of American nurses reported anxiety and depression during COVID-9 pandemic, respectively (35). Besides, the study from Iran found 38.8 and 37.4% nurses during COVID-19 had anxiety and depression symptoms (36). However, no study has reported the prevalence of female nurses' mental problems in the normalization of COVID-19 epidemic prevention and control. Moreover, in comparison to the prevalence of mental problems during the non-epidemic stage, our finding was higher (37–39), indicating although the mental health of female nurses has become better during the normalization stage, mental health problems still remain prevalent, suggesting special attention should be paid to female nurses.

Zhang et al. found that nurses experienced less psychological stress during the normalization of COVID-19 prevention and control (40). They pointed out that the reduction was related with the experience of fighting against COVID-19 epidemic, effective response to the epidemic and the stable condition of the COVID-19 epidemic. Therefore, female nurses in the present study reported less anxiety symptoms than those in the previous study. Female nurses need to contact with various patients or even access the blood samples during the outbreak of COVID-19, which may increase their risk of being infected by COVID-19 virus. Previous research confirmed that fear of being infected, or infecting others was associated with nurses' depression (41), which might explain why our participants had a more severe level of depression.

Moreover, we found insomnia, PTSD and marital status were associated factors affecting anxiety and depression of female nurses, which is consistent with previous literature (42–45). A review by Taylor et al. concluded that insomnia was a strong risk factor for depression and anxiety (46). Moreover, people with insomnia reported higher levels of anxiety and depression than those without insomnia and were 17.35 and 9.82 times as likely to have clinically significant anxiety and depression, respectively (47). Therefore, insomnia could predict mental health problems of female nurses. Earlier studies indicated that medical care workers were more likely to develop mental health problems due to their traumatic experience (48, 49). A systematic review by Naushad et al. implied that measures for medical care workers to prevent PTSD would be good for decreasing adverse psychological outcomes (44). Hence, PTSD symptom was an associated factor of mental health problems. In line with previous results (50–52), those who were unmarried had a higher level of depressive symptoms, and the possible explanation was that they might receive less support from family (42).

In a study focusing on mental health of medical staff in Xinjiang province of China based on the normalization of COVID-19 epidemic prevention and control (8). Researchers reported that being a nurse, poor health condition, living with elderly parents, less social support, and negative coping style were associated with experiencing worse mental health outcomes. Furthermore, Chen et al. conducted a cross-sectional study concerning potential impact factors of mental health among nurses under normalized COVID-19 pandemic prevention and control in Jiangsu province of China (10). They found that Having 11–15 years of working experience and being a fixed-term contract nurse were associated factors of nurses' mental health outcomes, while supporting-Wuhan working experience and having mental health preparation course training were protective factors. Our results revealed insomnia and PTSD symptom were associated factors of female nurses' anxiety and depression, while marital status was a protective factor of depression. We focused Chinese female nursing staff and expanded the associated factors of mental health in health-care workers under normalized COVID-19 pandemic prevention and control of China.

The results of the present study have profound implications for the amelioration of Chinese female nurses' mental health problems in the normalization of COVID-19 epidemic prevention and control practically. On the one hand, this study provided empirical data about prevalence of anxiety and depression among the female nursing staff under the regular COVID-19 epidemic prevention and control, suggesting the policy makers should pay more attention and design interventions to prevent mental problems of female nurses even under the normalization stage. On the other hand, our findings also detected associated factors influencing mental health of female nurses, which may provide potential preventive measures of anxiety and depression. Measures focusing on promoting sleep quality and decreasing stress from traumatic events such as meditation, music, mind-body bridging and yoga (53–56) must be taken to maintain good status of mental health among female nurses. Moreover, the unmarried female nursing staff needs more care from their colleagues, superiors and family members.

Several limitations in current study need to be mentioned. First of all, the cross-sectional design failed to confirm the causal relationship between insomnia, PTSD, marital status and mental problems. Longitudinal studies are needed in the future study to determine the casual associations between variables. Second, the participants were only from Jiangsu Province, China and were recruited through convenience sampling, which may limit the sample representativeness of the present study. It is better to recruit participants with random cluster sampling from other areas of China to increase the external validity of the present results. Third, our respondents completed the self-reported survey with mobile devices, which might lead to self-reported biases and social desirability response bias. Multi-informant measures are needed in further research to collect information from both self-report and other-report data with the purpose to avoid underestimation or overestimation of the associations. Fourth, the respondents of the present research were all female nurses, which limited the generalization of our results. Hence, our results can only be applied to female nursing staff. Moreover, it had to be noticed that the use of odds ratio may overestimate the association of associated factors with mental health of female nurses in our cross-sectional study.

## Conclusion

Our findings demonstrated that under the normalization of COVID-19 epidemic prevention and control, anxiety and depression were prevalent in Chinese female nurses, with a prevalence of 7.9 and 17.8%, respectively. Insomnia and PTSD were associated factors of mental health status, and being married was a protective factor of depression. Chinese hospital management should make effort to improve female nurses' sleep quality and reduce their subjective stress caused by traumatic events. Besides, more attention should be paid to the unmarried group.

## Data availability statement

The raw data supporting the conclusions of this article will be made available by the authors, without undue reservation.

## Ethics statement

The studies involving human participants were reviewed and approved by Naval Medical University. The patients/participants provided their written informed consent to participate in this study.

## Author contributions

XM, WDo, and JZ contributed to the writing of this article and the statistical analysis. TH leaded the whole study, including carrying out this study, and putting forward the study. FZ contributed to the final analyses and critical work on the final versions of the article. WDe and ZL edited and proofread the manuscript. All authors listed have made a substantial, direct, and intellectual contribution to the work and approved it for publication.
